# Negative Effects of *Phthorimaea absoluta*-Resistant Tomato Genotypes on the Zoophytophagous Biocontrol Agent, *Orius laevigatus* (Fieber) (Hemiptera: Anthocoridae)

**DOI:** 10.3390/insects14020160

**Published:** 2023-02-07

**Authors:** Megha Guruswamy, Murugan Marimuthu, Moshe Coll

**Affiliations:** 1 Department of Agricultural Entomology, Tamil Nadu Agricultural University, Coimbatore 641003, India; 2 Department of Entomology, Faculty of Agriculture, Food and Environment, The Hebrew University of Jerusalem, Rehovot 76100, Israel

**Keywords:** host plant resistance, biological control, tomato genotypes, tri-trophic interactions

## Abstract

**Simple Summary:**

We tested complex tri-trophic level interactions among a crop plant, a pest, and its natural enemy to maximize plant protection by compatible, combined contributions of crop resistance and biological control. Fitness components of the predatory bug *Orius laevigatus* were inferior to *Phthorimaea absoluta*-resistant tomato genotypes. These findings support the idea that biological control and crop resistance are incompatible in this system and highlight the need to consider biological control agents when developing crops for pest resistance. In sustainable integrated pest management systems, only the complementary actions of pest-resistant crops and biological controls could offer reliable and cost-effective plant protection.

**Abstract:**

Complex interactions between host plant resistance (HPR) and biological control agents, particularly omnivorous predators, can shape the outcome of an integrated pest management (IPM) program. However, such interactions are seldom explored during plant breeding programs. Therefore, in the present study, we compared the performance of the omnivorous biological control agent *Orius laevigatus* on six tomato genotypes with different levels of resistance to the tomato leaf miner *Phthorimaea absoluta*. We found that the *O. laevigatus* fitness components (i.e., egg deposition, egg hatching rate, and duration of egg, early nymphal, late nymphal stages, and their survival) were inferior on the wild resistant genotypes (LA 716 and LA 1777) in comparison to the resistant domesticated genotype EC 620343 and the susceptible genotypes (EC 705464 and EC 519819). It appears that the adverse effects of tomato genotypes on *O. laevigatus* are determined mainly by glandular and non-glandular trichome densities on the leaves. Comparison of *O. laevigatus* response to the tested tomato cultivars to that of *P. absoluta* revealed significant positive correlations in duration of the egg stages, development time of early and late larval stages, and overall immature mortality in both species. It appears, therefore, that defensive plant traits operate in a similar way on the pest and its predator in the system. Overall, the present study of the tomato-*P. absoluta*-*O. laevigatus* system provides experimental evidence for the need to optimize pest management by employing intermediate levels of crop resistance together with biological control agents.

## 1. Introduction

A wide range of defense mechanisms are employed by crop plants against herbivorous pests and diseases [1]. In turn, these defenses may interact with other plant protection agents in integrated pest management (IPM) systems [2]. As a result, interactions between biological control agents and host plant resistance (HPR) often shape the efficacy of sustainable IPM programs [3,4]. This is particularly important for biological control agents, such as predators and parasitoids. Yet, the outcome of such interactions depends on the operating direct and indirect effects and the mode of action of plant resistance traits on biological control agents as well as other biotic stressors [4,5,6].

Some resistance mechanisms that protect plants from herbivorous insects may harm or repel beneficial predatory insects, making HPR and biological control partially incompatible [7,8]. For example, plant resistance may have an adverse impact on natural enemies when toxic secondary plant compounds are passed on to natural enemies through the pest, resulting in prey-mediated indirect effects [9,10]. Furthermore, the effectiveness of natural enemies may be compromised by plant physical structures, such as leaf toughness, cuticle thickness, leaf waxy surface, and trichomes host plant-mediated direct effects [11]. In contrast, natural enemies often use herbivore-induced synomones emitted by crop plants to find and attack pests [12,13,14,15], and partial plant resistance may benefit natural enemies by reducing the growth rate of pests, thus making them susceptible to predation for a longer time [11,16]. Taken together, it is important to assess plant-pest-natural enemy interactions in crop protection programs when both host plant resistance and biological control are employed [17]. Yet, the negative effects of crop resistance traits on parasitoids and predators have largely been ignored during the development of insect-resistant crop varieties [7].

Tomato (*Solanum lycopersicum* L.) is the world’s most important vegetable crop [18]. However, its production is hampered by several biotic stressors, including insects, pathogens, and nematodes [19,20,21,22]. One of the major insect pests of tomato worldwide is the tomato leaf miner *Phthorimaea* (=*Tuta*) *absoluta* (Meyrick) (Lepidoptera: Gelechiidae), which feeds on leaf parenchyma at the larval stage, thus creating visible blotches [23]. The development of *P. absoluta*-resistant tomato cultivars is a sustainable alternative to insecticide application [1,9,24,25]. It is important, however, to make sure that the employment of resistant crops is compatible with the activity of natural enemies in the system [26]; there is ample evidence for the adverse effects of resistant genotypes on natural enemies [8,27]. Particularly susceptible to such negative effects of resistant crops are omnivorous predators that feed on plant materials in addition to prey [28]. However, the ability of omnivorous consumers to feed on plants and sustain viable populations when prey is scarce in the field is desirable for biological control because they can then retard crop recolonization by the pests [27,29,30]. 

In the present study, therefore, we tested the compatibility of *P. absoluta*-resistant tomato genotypes to *O. laevigatus.* For the following reasons, we decided to use *O. laevigatus* for the present study even though *Orius* performance on tomato plants is inferior compared to that on other crop plants (e.g., [31]) and compared to that of mirid bugs on tomato [32,33]: (i) *Orius* bugs are readily found in tomato fields, with more than 1700 published studies; (ii) the performance of mirid bugs, such as *Macrolophus* and *Nesidiocoris* species, on tomato has been studied extensively, with more than 5000 published papers; and (iii) we are aware of only two studies of the effect of tomato resistance on *Orius* bugs [27,34]. Yet, these studies explored the response of *O. insidiosus* and *O. sauteri*, respectively, to aphid- and whitefly-resistant tomato, and it is well established that plant resistance to sucking insects differs from that to chewing pests (*P. absoluta* in our study). 

The flower bug *Orius laevigatus* (Fieber) (Hemiptera: Anthocoridae) is an omnivorous generalist predator of tomato leaf miner and many other key agricultural pests [35]. However, the plant-feeding habits of omnivorous biological control agents, such as *O. laevigatus*, make them particularly susceptible to plant defenses. In addition, *O. laevigatus* inserts its eggs into leaf tissues, and its preference for oviposition sites is determined by plant characteristics [36,37]. It was therefore important to test the compatibility of *P. absoluta*-resistant tomato genotypes with *O. laevigatus.* Toward this goal, we compared fitness components, such as oviposition rate, survival, and development, of *O. laevigatus* on *P. absoluta*-resistant wild tomato genotypes, resistant domesticated genotypes, and susceptible domesticated genotypes. In addition, we correlated *O. laevigatus* performance with glandular and non-glandular trichome densities on the leaves. Finally, we correlated the performance of *O. laevigatus* and *P. absoluta* on these plant genotypes [38]. Results of this study provide direct assessment of the compatibility of insect-resistant crop genotypes and an omnivorous biological control agent. 

## 2. Materials and Methods

### 2.1. Plants 

Based on results obtained from an earlier screening for *P. absoluta* resistance in tomato [38], we selected six tomato genotypes (accessions) for the present study: two resistant wild genotypes (LA 716 and LA 1777), two resistant domesticated genotypes (EC 620343 and EC 631369), and two susceptible domesticated genotypes (EC 705464 and EC 519819). More than 15 seeds of each of the accessions were treated with 1% sodium hypochlorite (Y. S. Shion Ltd., Ashdod, Israel) for 30 min for sterilization and to break seed dormancy. After treatments, seeds were washed with running autoclaved water, and then seeds of each genotype were germinated collectively in a single plastic pot containing potting soil. The accessions were grown for three weeks in potting soil at 25 ± 2 °C in the greenhouse.

### 2.2. Insects 

A stock colony of *O. laevigatus* was established with about one thousand newly emerged adults obtained from BioBee (Sde Eliyahu, Israel). The bugs were reared in ventilated plastic boxes provided with *Ephestia kuehniella* eggs [39] attached to sticky Post-it^®^ Note (5 cm × 2 cm). Fresh tender geranium shoots were provided to the bugs every three days as water and food sources, as well as oviposition position medium. Egg-bearing shoots were collected and used to maintain the colony and for the experiments until adults survived [40]. The colony was maintained at 25 ± 2 °C and ca. 70% RH. 

### 2.3. Measure of O. laevigatus Fitness Components

**Figure 1:**
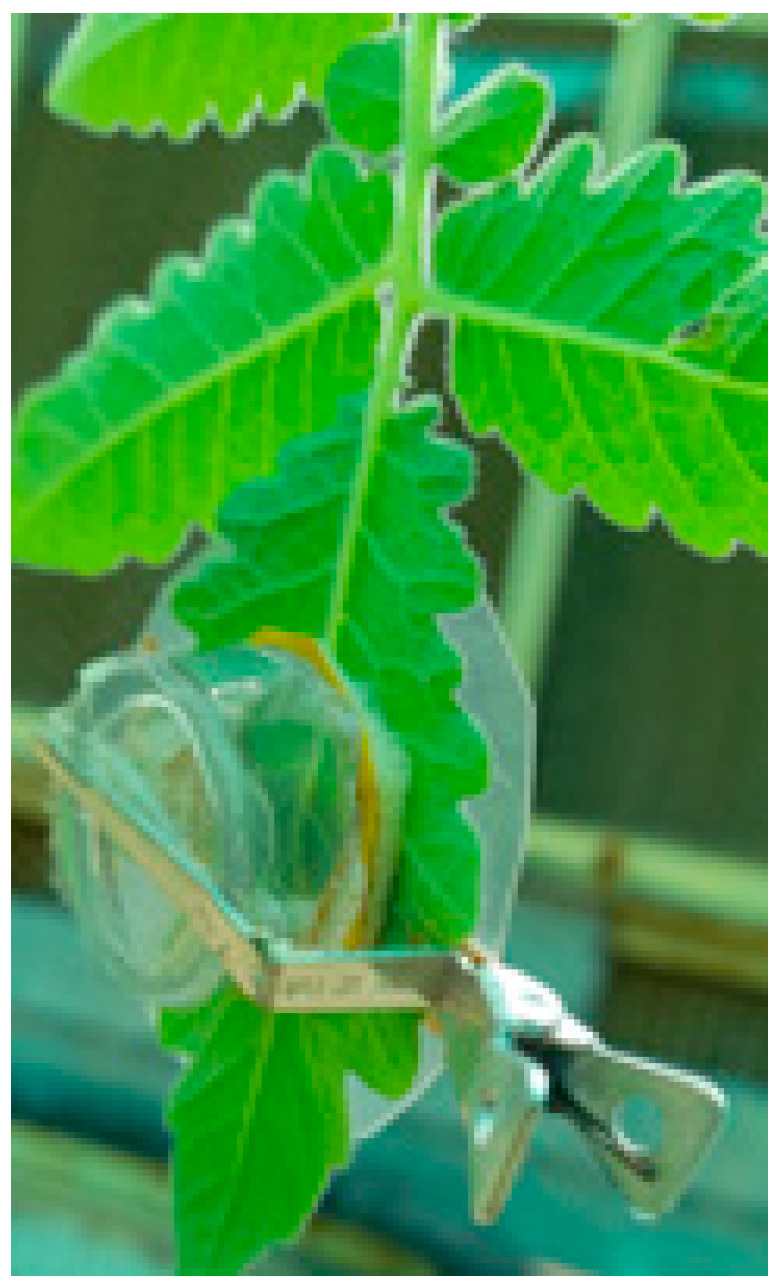
A clip leaf-cage with screened ventilated top.

Eight seedlings from each accession were transplanted individually in plastic pots and used as replicates for a total of 48 potted plants in a no-choice experiment in the greenhouse at 25 ± 2 °C. When the transplanted plants were 45 days old, 5-days old, single mated *O. laevigatus* females were placed individually in clip leaf-cage (2 cm in diameter) (Figure 1) that contained *E. kuehniella* eggs on a 0.25 cm^2^ Post-it^®^ Note. One leaf cage was set per plant, attached to the epical leaflet of the third leaf from the top.

Every 24 h and for 3 days, the caged females were moved to a new clip leaf-cage on another leaflet within the same plant (for a total of 72 h oviposition period), thus preventing exhaustion of oviposition sites and allowing us to monitor the newly emerged nymphs. Three newly emerged nymphs from each plant were caged individually on the same plant to prevent cannibalism. These nymphs were not provided with prey because we were interested in maximizing the effect of tomato genotypes on their fitness; this simulated a pest-scarcity situation in the field. We then monitored the nymphs daily until they molted to the adult stage or died. Therefore, three nymphs were monitored on each plant, replicated eight times per genotype for a total of 144 nymphs. Early and late instar mortality were determined from egg hatch to molting to the third instar, and from the molting to the third instar to adult eclosion, respectively. These data were used to calculate the duration of the egg, early, and late nymphal periods, total developmental time, early, and late-instar mortality, and total immature mortality. At the end of the experiment, the number of deposited eggs and the proportion of hatched eggs were determined for each clip cage using a dissecting stereoscope.

### 2.4. Measure of Leaf Trichome Density

We followed Maiti et al. [41] to compare trichome densities on the leaves of the six tomato genotypes used in the present study. Three 1 cm^2^ samples were collected at random from the third fully opened leaf from the top of each plant of the six genotypes. Each leaf sample was placed in 20 mL water at 85 °C for 15 min. The samples were then placed in 96% ethyl alcohol (Gadot group, Netanya, Israel) at 80 °C for 20 min. The alcohol soaking stage was repeated until all chlorophyll pigments were completely bleached off the leaves. To clear the leaf samples, 90% lactic acid (Fluka Analytical, Sigma-Aldrich Co., St. Louis MO, USA) was added to the test tubes containing leaf tissues, which were then sealed and kept at 85 °C for approximately 30–45 min. A drop of lactic acid was used to mount the cooled leaf samples onto microscope slides. An image analyzer was used under a stereo zoom microscope to count the number of glandular and non-glandular trichomes per cm^2^ leaf area.

### 2.5. Correlation between O. laevigatus and P. absoluta Performance Variables

In this part of the study, we correlated the performance of *P. absoluta* (data from a companion study [38]) and *O. laevigatus* (results of the present study) on the tested plant genotypes to gain insight into the resistance mechanisms that underlie the effects of tomato plants on these insects. We asked, do these plant genotypes have similar effects on the various fitness components of the two insects? We therefore correlated the egg period (the time required for an egg to hatch) of *P. absoluta* with that of *O. laevigatus*, developmental time of early stages of *P. absoluta* and *O. laevigatus*, developmental time of late stages of *P. absoluta* and *O. laevigatus*, and overall immature mortality of *P. absoluta* and *O. laevigatus*.

### 2.6. Data Analysis

The data were subjected to one-way analyses of variance (ANOVA; JMP 16). To meet the ANOVA assumptions, percent mortality data were square root-transformed before the analysis (JMP 16). Data that did not meet ANOVA assumptions (Levene test for homogeneity of variance) were analyzed using Wilcoxon and Kruskal-Wallis test (Chi-square test) (JMP 16). Means were compared using Tukey’s mean comparison test (JMP 16). The six tomato genotypes studied were used as data points in the correlation analyses of trichome vs. *O. laevigatus* performance, and *P. absoluta* vs. *O. laevigatus* performance (Pearson’s correlation; JMP 16). 

## 3. Results

### 3.1. Measure of O. laevigatus Fitness Components

**Figure 2:**
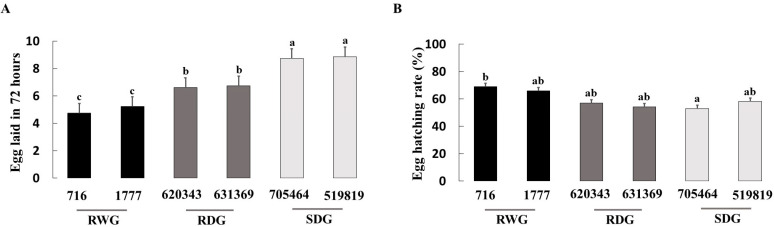
Mean number (±SE) of eggs laid by *Orius laevigatus* in 72 h (**A**) and egg hatching rate (**B**) on six tomato genotypes with different levels of resistance to *Phthorimaea absoluta*. Bars with shared letters do not differ significantly (one-way ANOVA; Tukey test; *p* ≤ 0.05). RWG = resistant wild genotypes; RDG = resistant domesticated genotypes; and SDG = susceptible domesticated genotypes.

**Figure 3:**
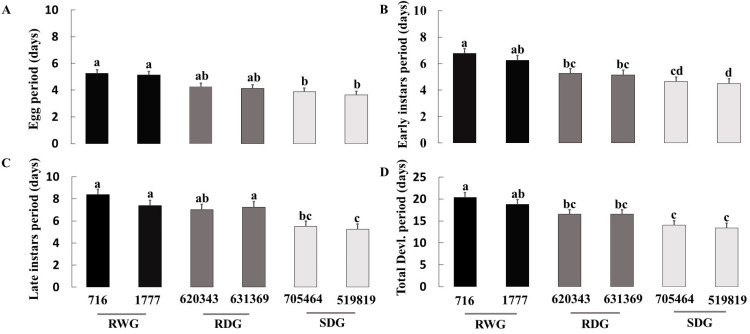
Mean (±SE) egg period (**A**), early instars period (**B**), late instars period (**C**), and total development period (**D**) of *Orius laevigatus* on six tomato genotypes of different levels of resistance to *Phthorimaea absoluta*. Bars with shared letters within the panel do not differ significantly (one-way ANOVA; Tukey’s test, *p* ≤ 0.05). RWG = resistant wild genotypes; RDG = resistant domesticated genotypes; and SDG = susceptible domesticated genotypes.

Significant differences were detected among the six tomato genotypes in the number of eggs laid by *O. laevigatus* females (F = 28.50; df = 5,42; *p* < 0.001; Figure 2A), egg hatching rate (F = 2.91; df = 5,42; *p* = 0.024; Figure 2B), duration of egg stage (F = 5.42; df = 5,42; *p* = 0.001; Figure 3A), duration of early nymphal stages (F = 6.60; df = 5,42; *p* < 0.001; Figure 3B), duration of late nymphal stages (F = 11.43; df = 5,42; *p* < 0.001; Figure 3C), duration of total nymphal stages (F = 16.64; df = 5,42; *p* < 0.001), and total immature development periods (F = 19.66; df = 5,42; *p* < 0.001; Figure 3D). 

**Figure 4:**
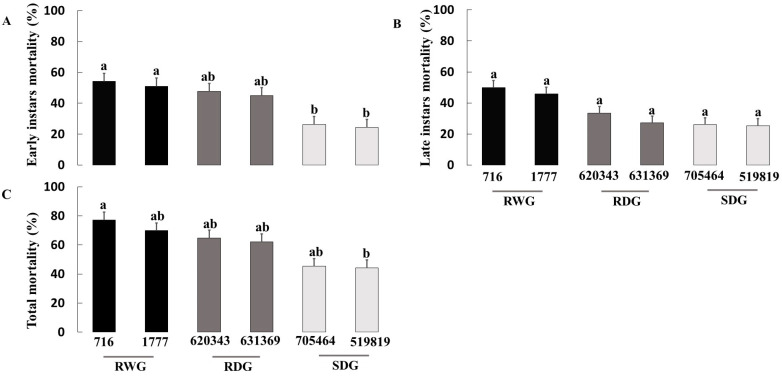
Mean mortality (% ± SE) of early instars (**A**), late instars (**B**), and total mortality to adulthood (**C**) of *Orius laevigatus* on six tomato genotypes with different levels of resistance to *Phthorimaea absoluta*. Bars with shared letters within the panel do not differ significantly (one-way ANOVA; Tukey test; *p* ≤ 0.05). RWG = resistant wild genotypes; RDG = resistant domesticated genotypes; and SDG = susceptible domesticated genotypes.

**Figure 5:**
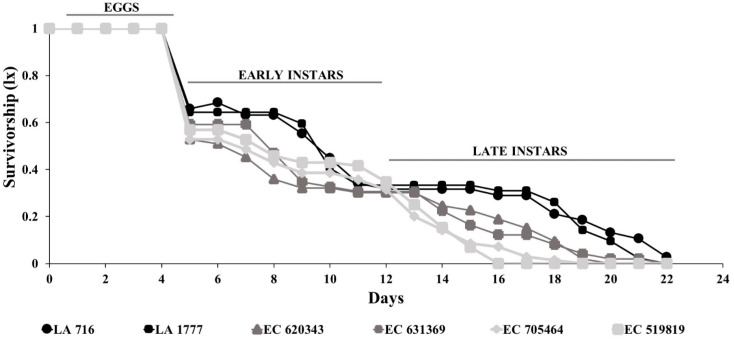
Survival curves of *Orius laevigatus* on *Phthorimaea absoluta*-resistant and susceptible tomato genotypes: Resistant wild genotypes (LA 716 and LA1777), resistant domesticated genotypes (EC 620343 and EC 631369), and susceptible domesticated genotypes (EC 705464 and EC 519819).

Tukey’s mean comparison test showed that all measured *O. laevigatus* fitness components were inferior on the wild resistance genotypes LA 716, followed by the other tested wild resistance genotypes LA 1777 and the domesticated resistant EC 620343. There was a significant correlation between the number of eggs laid by *O. laevigatus* females in 72 h and the proportion of egg hatched on each genotype (*r* = 0.75, *p* < 0.001). Likewise, tomato genotypes had a significant effect on the mortality rate at the early nymphal stage (χ^2^ = 22.2; df = 5; *p* = 0.001; Figure 4A) and total mortality (F = 2.19; df = 5; *p* = 0.024; Figure 4C), but not at the late nymphal stage (χ^2^ = 4.01; df = 5; *p* = 0.548; Figure 4B). Survival curves (Figure 5) indicate that most mortality occurs at the early developmental stages, and the survivorship was highest on the susceptible domesticated genotypes followed by resistant domesticated genotypes and resistant wild genotypes.

### 3.2. Trichome Density

**Figure 6:**
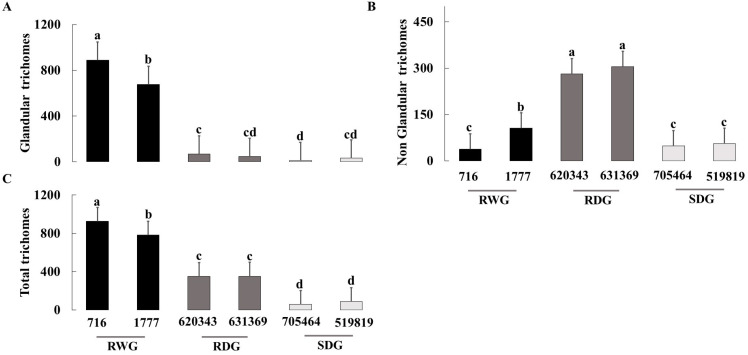
Mean density (number per 1 cm^2^ ± SE) of glandular (**A**), non-glandular (**B**), and total (**C**) trichomes on leaves of six tomato genotypes with different levels of resistance to *Phthorimaea absoluta*. Bars with shared letters within the panel do not differ significantly (one-way ANOVA; Tukey test; *p* ≤ 0.05). RWG = resistant wild genotypes; RDG = resistant domesticated genotypes; and SDG = susceptible domesticated genotypes.

Densities of glandular and non-glandular trichomes, and total leaf trichome density (glandular + non-glandular) differed significant among the six studied tomato genotypes (χ^2^ = 44.6, df = 5, *p* < 0.001; χ^2^ = 42.6, df = 5, *p* < 0.001; and χ^2^ = 44.2, df = 5, *p* < 0.001, respectively; Figure 5). The density of glandular trichomes and total leaf trichomes were highest on the wild resistance genotypes LA 716 and LA 1777, followed by the domesticated resistant EC 620343. In contrast, the density of non-glandular trichomes was highest on EC 631369, followed by EC 620343 and LA 1777 (Figure 6).

### 3.3. Effect of Trichome Density on O. laevigatus Survival

A significant positive correlation was found between the total glandular trichome density and early nymphal mortality and total immature mortality, and between total trichomes and early nymphal mortality and total immature mortality (Table 1).

### 3.4. Correlation between O. laevigatus and P. absoluta Performance Variables 

A significant positive correlation was detected between the duration of the *P. absoluta* egg period and that of *O. laevigatus*, the development time of the early stages of *P. absoluta* and *O. laevigatus*, the development time of the late stages of *P. absoluta* and *O. laevigatus*, and the total immature mortality of *P. absoluta* and *O. laevigatus* (Table 2).

## 4. Discussion

Our study focused on the effects of plant-resistant traits on an omnivorous biological control agent. In such systems, plant structural and chemical properties, such as trichome type and density and plant secondary compounds, may directly influence the oviposition site selection, development, and survival of players at the third trophic level [2,33,42]. Plant chemical composition may be particularly important to the performance of omnivorous consumers that feed on both prey and plant materials. Such omnivores may exhibit retarded development and impaired survival and fecundity when they feed on defended plants [34,43]. In addition, the efficacy of biological control agents may be inferior on crop plants that are defended by glandular trichomes [34,44]. It was therefore important to assess the direct effect of resistant crop genotypes on omnivorous predators of major pests in the system [2]. Our results show that the zoophytophagous predator *O. laevigatus* had the lowest egg hatch, longest development, lowest fecundity, and highest mortality on the tested wild tomato genotypes that exhibit the highest level of resistance to its prey *P. absoluta*. In addition, female bugs deposited significantly fewer eggs on *P. absoluta*-resistant genotypes than on susceptible plants. Yang et al. (2022) [34] reported similar results for *O. sauteri* on whitefly-resistant genotypes, suggesting that phytochemicals have a direct adverse effect on a beneficial species and phytochemical-mediated resistance can affect non-target insects. Additionally, they suggested that phytochemical-mediated resistance has a potential ecological impact. Given that mortality and glandular trichome density in our study have a negative correlation, phytochemicals may have an impact on the bug’s fitness.

Resistance properties of crop plants, such as glandular trichomes, often have a significant direct defensive role; they hinder oviposition and retard pest feeding and foraging. However, such plant defensive traits may also hamper indirect plant defenses provided by the natural enemies of these very pests [2,34,45,46,47,48,49]. Indeed, we found that the density of glandular trichomes was positively correlated not only with pest resistance (i.e., *P. absoluta*) but also with inferior performance of *O. laevigatus*. It seems that overall, *O. laevigatus* has a greater potential to rapidly build high population densities on *P. absoluta*-susceptible tomato genotypes due to its shortest life cycle, higher fecundity, and lowest mortality. The negative impact of trichomes on both *P. absoluta* and its predator may be magnified further, because higher trichome density on tomato stems may retard insect movement among plant parts [50,51,52]. Nonetheless, we found that the adverse effect of plant resistance on *O. laevigatus* mortality was more pronounced in early (first and second) than late instars (third–fifth). These results may be attributed to the greater dependency of the early stages of this omnivore on plant-provided nutrients compared to the later stages, which rely more on prey diet [53,54].

The positive correlations we detected between the duration of the egg period in *P. absoluta* and *O. laevigatus*, the development time of early and late immature stages of *P. absoluta* and *O. laevigatus*, and the total mortality of *P. absoluta* and *O. laevigatus* strongly suggest that similar plant defense mechanisms operate on the two players that occupy different trophic levels. This conclusion is supported by the plant-feeding habits of this omnivorous predator [30]. From an ecological standpoint, the most important concern is how plant feeding impacts omnivores’ ability to suppress prey on defended plants. Foraging theory holds that to meet their energy and nutrient requirements, omnivorous consumers should mix various plant and prey food sources in their diets [55]. There is much evidence that adding plant materials to their diet often enhances omnivores’ development [56], longevity [57], and fecundity [57,58,59] and alters their dispersal and distribution in the habitat [60,61]. The fact that omnivorous predators feed on a wide variety of foods and switch between prey and plant resources may also significantly influence their effects on herbivore populations [62,63,64,65]. For example, herbivores are released from predation when their omnivorous predators feed on high-quality plant materials, such as pollen [66,67,68,69]. Therefore, plant feeding may reduce prey consumption by individual omnivores. However, at the population level, incorporating plants in their diet often leads to higher predation because of enhanced reproduction, development, and survival that result in higher overall omnivore population size [30]. In turn, a decrease in plant quality is expected to increase predation by individual omnivores [65,70,71,72]. Therefore, it is important to quantify diet preferences by omnivorous predators and the impact that plant feeding has on their numerical response to changes in prey density [70,71,72].

The present study suggests that an intermediate, rather than maximal, level of HPR should be employed in integrated pest management programs in tomatoes [34,73]. Such optimal levels of resistance would reduce pest density to some extent but would not harm the natural enemies in the system, which would then further suppress pest populations. This was reported, for instance, for aphid populations on resistant *Vicia faba*; pest population was lowest even though *Coccinella septempunctata* predators had an increased development time and decreased fecundity on these plants [74]. Likewise, control of the Mexican bean beetle *Epilachna varivestis* was improved when resistant soybean cultivars were employed together with the biological control agent *Podisus maculiventris,* although this predator was less effective on resistant plants compared to susceptible plants [75]. Such an approach, therefore, would maximize plant protection by synergizing the desirable impacts of HPR and biological control. Further research on the tomato-*P. absoluta*-*O. laevigatus* system is needed to assess the individual and combined impacts of crop resistance and biological control on pest populations.

## 5. Conclusions

The tested fitness components of *O. laevigatus* were inferior to the *P. absoluta*-resistant tomato genotypes. These results suggest that biological control and crop resistance are incompatible in this system and provide strong evidence for the need to consider the action of biological control agents when breeding crops for pest resistance. Only compatible action of HPR and biological control could provide dependable and economical plant protection in sustainable integrated pest management systems. 

## Figures and Tables

**Table 1:** Correlation between trichome density and immature *O. laevigatus* mortality on six tomato genotypes.

Parameters	*r* Value	*p* Value
Glandular trichome vs. total mortality of *O. laevigatus*	0.47	0.003
Total trichomes vs. total mortality of *O. laevigatus*	0.49	<0.001
Total trichomes vs. early larval mortality of *O. laevigatus*	0.55	<0.001
Glandular trichome vs. early larval mortality of *O. laevigatus*	0.43	0.002

**Table 2:** Correlation between *O. laevigatus* and *P. absoluta* performance on six tomato genotypes.

Parameters	*r* Value	*p* Value
Egg period of *P. absoluta* vs. egg period of *O. laevigatus*	0.96	0.003
Development time of early stages of *P. absoluta* vs. development time of early stages of *O. laevigatus*	0.89	0.019
Development time of late stages of *P. absoluta* vs. development time of late stages of *O. laevigatus*	0.92	0.011
Total mortality of *P. absoluta* vs. total mortality of *O. laevigatus*	0.86	0.030

## Data Availability

From authors.

## References

[1] Stout M.J., Abrol D.P. (2014). Host-Plant Resistance in Pest Management. Integrated Pest Management.

[2] Peterson J.A., Ode P.J., Oliveira-Hofman C., Harwood J.D. (2016). Integration of plant defense traits with biological control of arthropod pests: Challenges and opportunities. Front. Plant. Sci..

[3] Bergman J.M., Tingey W.M. (1979). Aspects of interaction between plant genotypes and biological control. Bull. Entomol. Soc. Am..

[4] Verkerk R., Leather S., Wright D.J. (1998). The potential for manipulating crop–pest–natural enemy interactions for improved insect pest management. Bull. Entomol. Res..

[5] Gautam S., Mohankumar S., Kennedy J.S. (2016). Induced host plant resistance in cauliflower by *Beauveria bassiana*. J. Entomol. Zool. Stud..

[6] Prabhukarthikeyan R., Saravanakumar D., Raguchander T. (2014). Combination of endophytic *Bacillus* and *Beauveria* for the management of Fusarium wilt and fruit borer in tomato. Pest Manag. Sci..

[7] Groot A.T., Dicke M. (2001). Transgenic Crops in an Agro-Ecological Context: Multitrophic Aspects of Insect-Resistant Plants.

[8] Tandon P., Upadhyay R.K., Mukerji K.G., Chamola B.P. (2001). Negative Aspects of Interaction between Host Plant Resistance and Biological Control and Its Implication in Integrated Pest Management of Crops. Biocontrol Potential and its Exploitation in Sustainable Agriculture.

[9] Hare J.D., Fritz R.S., Simms E.L. (1992). Effects of Plant Variation on Herbivore-Natural Enemy Interaction. Plant Resistance to Herbivores and Pathogens: Ecology, Evolution, and Genetics.

[10] Harvey J.A., Van Dam N.M., Gols R. (2003). Interactions over four trophic levels: Foodplant quality affects development of a hyperparasitoid as mediated through a herbivore and its primary parasitoid. J. Anim. Ecol..

[11] Price P.W., Bouton C.E., Gross P., McPheron B.A., Thompson J.N., Weis A.E. (1980). Interactions among three trophic levels: Influence of plants on interactions between insect herbivores and natural enemies. Annu. Rev. Ecol. Syst..

[12] Vet L.E., Dicke M. (1992). Ecology of infochemical use by natural enemies in a tritrophic context. Annu. Rev. Entomol..

[13] Dicke M., van Poecke R.M., de Boer J.G. (2003). Inducible indirect defence of plants: From mechanisms to ecological functions. Basic Appl. Ecol..

[14] Arimura G.I., Kost C., Boland W. (2005). Herbivore-induced, indirect plant defences. Biochim. Biophys. Acta (BBA) Mol. Cell Biol. Lipids..

[15] D’Alessandro M., Turlings T.C. (2006). Advances and challenges in the identification of volatiles that mediate interactions among plants and arthropods. Analyst.

[16] Feeny P., Wallace J.M., Mansell R.L. (1976). Plant Apparency and Chemical Defense. Biochemical Interaction between Plants and Insects.

[17] Thomas M., Waage J. (1996). Interaction between Host-Plant Resistance and Biological Control. Integration of Biological Control and Host Plant Resistance Breeding: A Scientific and Literature Review.

[18] Kimura S., Sinha N. (2008). Tomato (*Solanum lycopersicum*): A model fruit-bearing crop. Cold Spring Harb. Protoc..

[19] Balaji D.R., Jeyarani S., Ramaraju K., Mohankumar S., Shanmugam (2018). Occurrence of South American tomato pinworm, *Tuta absoluta* (Meyrick). (Lepidoptera: Gelechiidae): An invasive pest in Tamil Nadu, India. J. Entomol. Zool. Stud..

[20] Marchant W.G., Gautam S., Dutta B., Srinivasan R. (2022). Whitefly-Mediated Transmission and Subsequent Acquisition of Highly Similar and Naturally Occurring Tomato Yellow Leaf Curl Virus Variants. Phytopathology.

[21] Panno S., Davino S., Caruso A.G., Bertacca S., Crnogorac A., Mandić A., Noris E., Matić S. (2021). A review of the most common and economically important diseases that undermine the cultivation of tomato crop in the Mediterranean basin. Agronomy.

[22] Jothi G., Pugalendhi S., Poornima K., Rajendran G. (2003). Management of root-knot nematode in tomato *Lycopersicon esculentum*, Mill., with biogas slurry. Bioresour. Technol..

[23] Vargas C. (1970). Observaciones sobre la biologia y enemigos naturales de la polilla del tomate, *Gnorimoschema absoluta* (Meyrick).(Lep. Gelechiidae). Idesia.

[24] Smith C.M. (2005). Plant Resistance to Arthropods: Molecular and Conventional Approaches.

[25] Nombela G., Muñiz M., Stansly P., Naranjo S. (2009). Host plant resistance for the management of *Bemisia tabaci*: A multi-crop survey with emphasis on tomato. Bemisia: Bionomics and Management of a Global Pest.

[26] Bottrell D.G., Barbosa P., Gould F. (1998). Manipulating natural enemies by plant variety selection and modification: A realistic strategy. Annu. Rev. Entomol..

[27] Pallipparambil G.R., Sayler R.J., Shapiro J.P., Thomas J.M., Kring T.J., Goggin F.L. (2015). Mi-1.2, an R gene for aphid resistance in tomato, has direct negative effects on a zoophytophagous biocontrol agent, *Orius insidiosus*. J. Exp. Bot..

[28] Bottega D.B., de Souza B.H.S., Rodrigues N.E.L., Eduardo W.I., Barbosa J.C., Júnior A.L.B. (2017). Resistant and susceptible tomato genotypes have direct and indirect effects on *Podisus nigrispinus* preying on *Tuta absoluta* larvae. Biol. Control.

[29] Armer C.A., Wiedenmann R.N., Bush D.R. (1998). Plant feeding site selection on soybean by the facultatively phytophagous predator *Orius insidiosus*. Entomol. Exp. Appl..

[30] Eubanks M.D., Styrsky J.D., Wackers F.L., van Rijn P.C.J., Bruin J. (2005). Effects of plant feeding on the performance of omnivorous predators. Plant-Provided Food for Carnivorous Insects: A Protective Mutualism and Its Applications.

[31] Coll M., Ridgway R.L. (1995). Functional and numerical responses of *Orius insidiosus* (Heteroptera: Anthocoridae) to its prey in different vegetable crops. Ann. Entomol. Soc. Am..

[32] Bueno V.H., Lins J.C., Silva D.B., van Lenteren J.C. (2019). Is predation of *Tuta absoluta* by three Neotropical mirid predators affected by tomato lines with different densities in glandular trichomes. Arthropod Plant Interact..

[33] Wheeler A.G., Krimmel B.A. (2015). Mirid (Hemiptera: Heteroptera) specialists of sticky plants: Adaptations, interactions, and ecological implications. Annu. Rev. Entomol..

[34] Yang F., Zhang X., Shen H., Xue H., Tian T., Zhang Q., Hu J., Tong H., Zhang Y., Su Q. (2022). Flavonoid-producing tomato plants have a direct negative effect on the zoophytophagous biological control agent *Orius sauteri*. Insect Sci..

[35] Van Lenteren J.C., Alomar O., Ravensberg W.J., Urbaneja A., Gullino M.L., Albajes R., Nicot P.C. (2020). Biological Control Agents for Control of Pests in Greenhouses. Integrated Pest and Disease Management in Greenhouse Crops.

[36] Tan X.-L., Wang S., Liu T.X. (2014). Acceptance and suitability of four plant substrates for rearing *Orius sauteri* (Hemiptera: Anthocoridae). Biocontrol Sci. Technol..

[37] Zhang L., Qin Z., Liu P., Yin Y., Felton G.W., Shi W.J. (2021). Influence of plant physical and anatomical characteristics on the ovipositional preference of *Orius sauteri* (Hemiptera: Anthocoridae). Insects.

[38] Guruswamy M., Marimuthu M., Coll M. (2023). Life table analyses for the Tomato Leaf Miner, *Tuta absoluta* (Meyrick) (Lepidoptera: Gelechiidae): Effects of plant genotype. Pest Manag. Sci..

[39] Pehlivan S. (2021). Influence of the eggs of *Ephestia kuehniella* (Lepidoptera: Pyralidae) reared on different diets on the performance of the predatory bug *Orius laevigatus* (Hemiptera: Anthocoridae). Eur. J. Entomol..

[40] De Clercq P., Coudron T.A., Riddick E.W., Morales-Ramos J.A., Rojas G., Shapiro D.I., Morales-Ramos J.A., Rojas M.G., Shapiro-Ilan D. (2014). Production of Heteropteran Predators. Mass Production of Beneficial Organisms.

[41] Maiti R., Bidinger F., Reddy K.S., Gibson P., Davis J. (1980). Nature and Occurrence of Trichomes in Sorghum Lines with Resistance to the Sorghum Shootfly.

[42] Inbar M., Gerling D. (2008). Plant-mediated interactions between whiteflies, herbivores, and natural enemies. Annu. Rev. Entomol..

[43] Styrsky J.D., Kaplan I., Eubanks M.D. (2006). Plant trichomes indirectly enhance tritrophic interactions involving a generalist predator, the red imported fire ant. Biol. Control.

[44] Kennedy G.G. (2003). Tomato, pests, parasitoids, and predators: Tritrophic interactions involving the genus *Lycopersicon*. Annu. Rev. Entomol..

[45] Riddick E.W., Simmons A.M. (2014). Do plant trichomes cause more harm than good to predatory insects. Pest Manage. Sci..

[46] Paspati A., Rambla J.L., Gresa M.P.L., Arbona V., Gómez-Cadenas A., Granell A., González-Cabrera J., Urbaneja A. (2021). Tomato trichomes are deadly hurdles limiting the establishment of *Amblyseius swirskii* Athias-Henriot (Acari: Phytoseiidae). Biol. Control.

[47] Tian D., Tooker J., Peiffer M., Chung S.H., Felton G.W. (2012). Role of trichomes in defense against herbivores: Comparison of herbivore response to woolly and hairless trichome mutants in tomato (*Solanum lycopersicum*). Planta.

[48] Lucini T., Faria M.V., Rohde C., Resende J.T.V., de Oliveira J.R.F. (2015). Acylsugar and the role of trichomes in tomato genotypes resistance to *Tetranychus urticae*. Arthropod Plant Interact..

[49] Bitew M.K. (2018). Significant role of wild genotypes of tomato trichomes for *Tuta absoluta* resistance. J. Plant Genet. Breed..

[50] Simmons A.T., Gurr G.M. (2005). Trichomes of *Lycopersicon* species and their hybrids: Effects on pests and natural enemies. Agric. For. Entomol..

[51] Simmons A.T., Gurr G.M. (2006). The effect on the biological control agent *Mallada signata* of trichomes of F1 *Lycopersicon esculentum* × *L. cheesmanii* f. *minor* and *L. esculentum* × *L. pennellii* hybrids. Biol. Control.

[52] Maiti R., Satya P., Rajkumar D., Ranaswamy A. (2012). Anatomical changes in crops under adaptation. Crop Plant Anatomy.

[53] Coll M. (1996). Feeding and ovipositing on plants by an omnivorous insect predator. Oecologia.

[54] Barber G.W. (1936). Orius Insidiosus (Say), an Important Natural Enemy of the Corn Ear Worm.

[55] Agrawal A.A., Klein C.N. (2000). What omnivores eat: Direct effects of induced plant resistance on herbivores and indirect consequences for diet selection by omnivores. J. Anim. Ecol..

[56] Perdikis D., Lykouressis D. (2000). Effects of various items, host plants, and temperatures on the development and survival of *Macrolophus pygmaeus* Rambur (Hemiptera: Miridae). Biol. Control.

[57] Lee J.C., Heimpel G.E. (2008). Floral resources impact longevity and oviposition rate of a parasitoid in the field. J. Anim. Ecol..

[58] Nomikou M., Janssen A., Schraag R., Sabelis M.W. (2002). Phytoseiid predators suppress populations of *Bemisia Tabaci* on cucumber plants with alternative food. Exp. Appl. Acarol..

[59] Perdikis D.C., Lykouressis D.P. (2002). Life table and biological characteristics of *Macrolophus Pygmaeus* when feeding on *Myzus Persicae* and *Trialeurodes Vaporariorum*. Entomol. Exp. Appl..

[60] Eubanks M.D., Denno R.F. (1999). The ecological consequences of variation in plants and prey for an omnivorous insect. Ecology.

[61] Wanner H., Gu H., Dorn S. (2006). Nutritional value of floral nectar sources for flight in the parasitoid wasp, *Cotesia Glomerata*. Physiol. Entomol..

[62] Snyder W.E., Wise D.H. (2001). Contrasting trophic cascades generated by a community of generalist predators. Ecology.

[63] Finke D.L., Denno R.F. (2002). Intraguild predation diminished in complex-structured vegetation: Implications for prey suppression. Ecology.

[64] Bruno J.F., O’Connor M.I. (2005). Cascading effects of predator diversity and omnivory in a marine food web. Ecol. Lett..

[65] Eubanks M.D., Denno R.F. (2000). Host plants mediate omnivore–herbivore interactions and influence prey suppression. Ecology.

[66] Janssen A., Willemse E., Van Der Hammen T. (2003). Poor host plant quality causes omnivore to consume predator eggs. J. Anim. Ecol..

[67] Badii M.H., Hernández-Ortiz E., Flores A.E., Landeros J.A. (2004). Prey stage preference and functional response of *Euseius Hibisci* to *Tetranychus Urticae* (Acari: Phytoseiidae, Tetranychidae). Exp. Appl. Acarol..

[68] Spellman B., Brown M., Mathews C.R. (2006). Effect of floral and extrafloral resources on predation of *Aphis spiraecola* by *Harmonia axyridis* on apple. BioControl.

[69] Robinson K.A., Jonsson M., Wratten S.D., Wade M.R., Buckley H.L. (2008). Implications of floral resources for predation by an omnivorous lacewing. Basic Appl. Ecol..

[70] Coll M., Lundgren J.G. (2009). Feeding on Non-Prey Resources by Natural Enemies. Relationships of Natural Enemies and Non-Prey Foods.

[71] Gillespie D., McGregor R.R. (2000). The functions of plant feeding in the omnivorous predator *Dicyphus hesperus*: Water places limits on predation. Ecol. Entomol..

[72] Ågren G.I., Stenberg J.A., Björkman C. (2012). Omnivores as plant bodyguards—A model of the importance of plant quality. Basic Appl. Ecol..

[73] van Emden H.F. (1995). Host plant-aphidophaga interactions. Agric. Ecosys. Environ..

[74] Shannag H., Obeidat W.M. (2008). Interaction between plant resistance and predation of *Aphis fabae* (Homoptera: Aphididae) by *Coccinella septempunctata* (Coleoptera: Coccinellidae). Ann. Appl. Biol..

[75] Bartlett R. (2008). Negative interactions between chemical resistance and predators affect fitness in soybeans. Ecol. Entomol..

